# Supplementary material for: Facilitation of sensorimotor temporal recalibration mechanisms by cerebellar tDCS in patients with schizophrenia spectrum disorders and healthy individuals
Source: Sci Rep. 2024 Feb 1;14:2627. doi: 10.1038/s41598-024-53148-3 (PMC10830570; doi:10.1038/s41598-024-53148-3)
Supplement: Supplementary file 1 — Supplementary Information. [file 41598_2024_53148_MOESM1_ESM.docx]

**Supplementary material** for the research article: “Facilitation of temporal recalibration mechanisms by cerebellar tDCS in patients with schizophrenia spectrum disorders and healthy individuals”

# **S1. Sample characteristics**

Initially, 24 patients with SSD were included in the study, but two of them dropped out before finishing all sessions due to a loss of interest in participating. The final samples of HC and SSD were matched in terms of sex and the level of education (see **Supplementary Table 1**). There were no significant age difference between the groups [independent-samples t-test: *t*(40) = .435, *p* = .666, *d* = .134].

Before the first tDCS session, patients were invited to an additional session during which their diagnosis was verified with the Structured Clinical Interview for DSM-IV (SCID).^1^ Furthermore, additional clinical scales and neuropsychological tests were applied during this session (results are summarized in **Supplementary Table 1**). For HC, the same neuropsychological tests were applied during the tDCS sessions.

**Supplementary Table 1. Demographics and clinical characteristics.**

|  | **SSD**  (N = 22) | **HC**  (N = 20) | |
| --- | --- | --- | --- |
| ***Demographics*** |  |  | |
| Male/Female | 11/11 | 10/10 | |
| Age | 35.636 +/- 10.363 | 37.050 +/- 10.699 | |
| Higher Education | 13 | 14 | |
| ***Antipsychotic medication*** |  |  | |
| None | 4 | 20 | |
| FGA | 2^a^ | - | |
| SGA | 18 | - | |
| ***Neuropsychological tests*** |  |  | |
| Attention |  |  | |
| d2 score | 163.23 +/- 39.43 | 175.47 +/- 44.24^b^ | |
| Executive functions |  |  | |
| TMT-A (sec.) | 26.76 +/- 7.90 | 23.94 +/- 9.80 | |
| TMT-B (sec.) | **72.32 +/- 22.56** | **54.52 +/- 22.90** | |
| Short term memory |  |  | |
| WAIS: FS score | 7.50 +/- 1.44 | 7.65 +/- 1.75 | |
| WAIS: BS score | 6.14 +/- 1.21 | 6.60 +/- 1.43 | |
| ***Clinical scales*** |  |  | |
| SAPS score | 18.04 +/- 13.27 |  |  |
| SANS score | 13.64 +/- 11.17 |  |  |
| BDI score | 0.62 +/- 0.53 |  |  |
| GAF score | 60.50 +/- 15.91 |  |  |
| SOFAS score | 78.0 +/- 14.01 |  |  |

*Note.* FGA = First generation antipsychotics, SGA = Second generation antipsychotics, d2: d2 test of attention,^2^ TMT: Trial Making Test,^3^ WAIS: Wechsler Adult Intelligence Scale,^4^ FS: Forward span, BS: Backward span, SAPS: Scale for the Assessment of Positive Symptoms,^5^ SANS: Scale for the Assessment of Negative Symptoms,^6^ BDI: Beck Depression Inventory,^7^ GAF: Global Assessment of Functioning,^8^ SOFAS: Social and Occupational Functioning Assessment Scale.^9^ For continuous variables the mean +/- standard deviation is displayed. Significant differences between HC and SSD are presented with bold values [TMT-B: *t*(40) = -2.536, *p* = .015, *d* = -.784; there were no group differences for any other comparisons: all *p* > .260]. ^a^Two patients were medicated with both FGA and SGA. ^b^N = 19.

**S2. Training Procedure**

To ensure that button presses were correctly performed and that participants were familiar with the task, all participants went through a training procedure during the first tDCS session prior to the stimulation. They were trained to let their finger be moved by the button device in passive condition without applying any counter-pressure. They were also trained to perform the button presses with the correct timing, i.e., in intervals of approx. 800ms in adaptation phases and for a duration of approx. 500ms (in both adaptation and test phases). The button presses were chosen to last for 500ms to ensure that stimuli were presented before the upward movement of the button for all test delay levels (the max. delay was 417ms), since it may interfere with delay detection. Even though participants were trained to perform the active button presses with the parameters described above, further measures were taken during the experiment to assure comparable button press parameters for active and passive movement conditions: Passive button press intervals and durations adapted to the mean of the respective preceding active conditions. Adaptation phases always terminated automatically after nine button presses in each of the two parts. If participants completed the button presses too fast in active conditions during a part of the adaptation phase (i.e., faster than 8000ms), the jitter between the adaptation phases (when moving too fast in the first part) or the instruction text for the following test phase (when moving too fast in the second part) were extended by the remaining time. Additionally, participants trained the test phases for each experimental condition, once with no delay and once with the maximum delay between button movement and outcome (417ms) to familiarize with the stimuli and the delays. During the training of test trials, they received feedback about the actual presence of a delay. Responses about the presence of a delay were instructed to be given as accurately, but not as fast as possible. Lastly, they went through in a 10-minute training of the experiment to further familiarize with the task.

**S3. Overview of all main and interaction effects for the main analysis**

A mixed ANOVA with the between-participants factor *group* and the within-participants factors *stimulation*, *test modality*, *movement type*, and *adaptation delay* was used to assess the impact of these variables on the percentage of detected delays during the test phases. Of main importance were main and interaction effects including the factor *adaptation delay* since modulations of delay detection performances were expected to occur after exposure to the delayed (200ms) vs. undelayed tone during adaptation phases. **Supplementary Table 2** provides an overview of all effects of this analysis. Please refer to the main manuscript for an interpretation and discussion of the relevant effects. Furthermore, note that excluding the first four patients for which the adaptation delay had been set to 150ms instead of 200ms, did not yield group differences in the overall TRE [*t*(36) = 1.508, *p* = .140, *d* = .490, two-sided], indicating that these patients did not fundamentally affect the reported temporal recalibration results.

**Supplementary Table 2. Results of the ANOVA testing for differences in delay detection performances between the experimental conditions**

| **Effect** | **Sum of Squares** | **df** | **Mean Square** | **F-val.** | **p-val.** | **η_p_^2^** |  |
| --- | --- | --- | --- | --- | --- | --- | --- |
| **Group** | 1042.879 | 1 | 1042.879 | 0.239 | 0.628 | 0.006 |  |
| Residuals | 174743,407 | 40 | 4368.585 |  |  |  |  |
| **Stimulation** | 1889.121 | 3 | 629.707 | 3.228 | 0.025 | 0.075 |  |
| **Stimulation * Group** | 579.187 | 3 | 193.062 | 0.990 | 0.400 | 0.024 |  |
| Residuals | 23411.494 | 120 | 195.096 |  |  |  |  |
| **Test modality** | 276.511 | 1 | 276.511 | 1.072 | 0.307 | 0.026 |  |
| **Test modality * Group** | 300.903 | 1 | 300.903 | 1.167 | 0.286 | 0.028 |  |
| Residuals | 10313.270 | 40 | 257.832 |  |  |  |  |
| **Movement type** | 8490.601 | 1 | 8490.601 | 23.817 | < .001 | 0.373 |  |
| **Movement type * Group** | 15.472 | 1 | 15.472 | 0.043 | 0.836 | 0.001 |  |
| Residuals | 14259.492 | 40 | 356.487 |  |  |  |  |
| **Adaptation delay** | 3381.799 | 1 | 3381.799 | 14.033 | < .001 | 0.260 |  |
| **Adaptation delay * Group** | 64.406 | 1 | 64.406 | 0.267 | 0.608 | 0.007 |  |
| Residuals | 9639.504 | 40 | 240.988 |  |  |  |  |
| **Stimulation * Test modality** | 41.766 | 3 | 13.922 | 0.487 | 0.692 | 0.012 |  |
| **Stimulation * Test modality * Group** | 20.466 | 3 | 6.822 | 0.239 | 0.869 | 0.006 |  |
| Residuals | 3429.039 | 120 | 28.575 |  |  |  |  |
| **Stimulation * Movement type** | 498.654 | 3 | 166.218 | 2.905 | 0.038 | 0.068 |  |
| **Stimulation * Movement type * Group** | 1.018 | 3 | 0.339 | 0.006 | 0.999 | 1.483e -4 |  |
| Residuals | 6865.225 | 120 | 57.210 |  |  |  |  |
| **Test modality * Movement type** | 3085.947 | 1 | 3085.947 | 57.555 | < .001 | 0.590 |  |
| **Test modality * Movement type * Group** | 4.002 | 1 | 4.002 | 0.075 | 0.786 | 0.002 |  |
| Residuals | 2144.706 | 40 | 53.618 |  |  |  |  |
| **Stimulation * Adaptation delay** | 442.776 | 3 | 147.592 | 2.800 | 0.043 | 0.065 |  |
| **Stimulation * Adaptation delay * Group** | 28.934 | 3 | 9.645 | 0.183 | 0.908 | 0.005 |  |
| Residuals | 6324.609 | 120 | 52.705 |  |  |  |  |
| **Test modality * Adaptation delay** | 398.065 | 1 | 398.065 | 9.229 | 0.004 | 0.187 |  |
| **Test modality * Adaptation delay * Group** | 8.787 | 1 | 8.787 | 0.204 | 0.654 | 0.005 |  |
| Residuals | 1725.286 | 40 | 43.132 |  |  |  |  |
| **Movement type * Adaptation delay** | 289.127 | 1 | 289.127 | 8.762 | 0.005 | 0.180 |  |
| **Movement type * Adaptation delay * Group** | 74.661 | 1 | 74.661 | 2.263 | 0.140 | 0.054 |  |
| Residuals | 1319.897 | 40 | 32.997 |  |  |  |  |
| **Stimulation * Test modality * Movement type** | 81.283 | 3 | 27.094 | 1.558 | 0.203 | 0.037 |  |
| **Stimulation * Test modality * Movement type * Group** | 17.785 | 3 | 5.928 | 0.341 | 0.796 | 0.008 |  |
| Residuals | 2086.397 | 120 | 17.387 |  |  |  |  |
| **Stimulation * Test modality * Adaptation delay** | 117.841 | 3 | 39.280 | 1.425 | 0.239 | 0.034 |  |
| **Stimulation * Test modality * Adaptation delay * Group** | 18.754 | 3 | 6.251 | 0.227 | 0.878 | 0.006 |  |
| Residuals | 3307.155 | 120 | 27.560 |  |  |  |  |
| **Stimulation * Movement type * Adaptation delay** | 15.263 | 3 | 5.088 | 0.131 | 0.941 | 0.003 |  |
| **Stimulation * Movement type * Adaptation delay * Group** | 19.708 | 3 | 6.569 | 0.169 | 0.917 | 0.004 |  |
| Residuals | 4656.530 | 120 | 38.804 |  |  |  |  |
| **Test modality * Movement type * Adaptation delay** | 460.901 | 1 | 460.901 | 7.781 | 0.008 | 0.163 |  |
| **Test modality * Movement type * Adaptation delay * Group** | 33.242 | 1 | 33.242 | 0.561 | 0.458 | 0.014 |  |
| Residuals | 2369.495 | 40 | 59.237 |  |  |  |  |
| **Stimulation * Test modality * Movement type * Adaptation delay** | 179.676 | 3 | 59.892 | 3.343 | 0.022 | 0.077 |  |
| **Stimulation * Test modality * Movement type * Adaptation delay * Group** | 5.751 | 3 | 1.917 | 0.107 | 0.956 | 0.003 |  |
| Residuals | 2149.907 | 120 | 17.916 |  |  |  |  |

*Note.* N_HC_ = 20, N_SSD_ = 22

**S4. Stimulation side effects**

After each session, participants reported on a custom-designed questionnaire whether they perceived any side effects due to the tDCS stimulation [on a scale from one (no side effect) to five (strong side effect) for 28 items]. To test for potential differences in perceived stimulation side effects between groups and stimulation conditions, we conducted a mixed ANOVA with the between-participants factor *group* and the within-participants factor *stimulation*. A full overview of the results is displayed in **Supplementary Table 3**. There was a significant main effect of *group* indicating that patients with SSD (*Mean* = 1.608, *SD* = ﻿.441) reported stronger perceived side effects than HC (*Mean* = ﻿1.229, *SD* = ﻿.281). Importantly, since there were no group differences in the TRE or in the impact of tDCS on the TRE in our study, this difference in perceived stimulation side effects should not have influenced our reported main results. There was no significant main effect of *stimulation* and no interaction of the factors *group* and *stimulation*, indicating that the amount of perceived side effects did not differ between the different stimulation conditions.

**Supplementary Table 3. Results of the ANOVA testing for differences in perceived stimulation side effects.**

| **Effect** | **Sum of Squares** | **df** | **Mean Square** | **F-val.** | **p-val.** | **η_p_^2^** |
| --- | --- | --- | --- | --- | --- | --- |
| **Group** | 6.030 | 1 | 6.030 | 18.459 | < .001 | .316 |
| Residuals | 13.067 | 40 | .327 |  |  |  |
| **Stimulation** | .357 | 3 | .119 | 1.535 | .209 | .037 |
| **Stimulation * Group** | .421 | 3 | .140 | 1.810 | .149 | .043 |
| Residuals | 9.307 | 120 | .078 |  |  |  |

*Note.* N_HC_ = 20, N_SSD_ = 22

**S5. Group-dependent differences in delay detection performance between active and passive conditions**

The rationale of the present study was based on the established finding that patients with SSD show impairments in predicting the sensory outcomes of self-generated actions which manifests in reduced perceptual differences between actively vs. passively elicited stimuli in SSD compared to HC.^10–13^ Here, we investigated whether this impairment may partly be attributed to dysfunctional sensorimotor temporal recalibration mechanisms. Thus, firstly, the question arises as to whether there was such a general impairment in predicting sensory action-outcomes in patients, meaning whether they showed a reduced difference in the processing of actively vs. passively generated stimuli in our study.

Both groups detected more delays in active compared to passive conditions. According to paired-samples t-tests, this difference was significant in HC [*Mean difference* = 6.591,
*SD* = 6.561, *t*(19) = 4.493, *p* < .001, *d* = 1.005, two-sided], but failed to reach significance for SSD [*Mean difference* = 3.635, *SD* = 9.135, *t*(21) = 1.866, *p* = .076, *d* = .398, two-sided]. Nonetheless, this group difference appeared to be not strong enough or the variance within the groups might have been too large to lead to a significant *group* x *movement type* interaction in the ANOVA reported in the main manuscript.

However, since it may be reasonable to assume that the active-passive difference in delay detection occurs for certain delay levels only, we also conducted a generalized estimating equations (GEE) analysis using IBM SPSS Statistics (Version 27.0) with the active-passive difference in the percentage of detected delays as dependent variable, which was computed separately for each of the six delay levels used during the test phases. An AR (1) working correlation structure and robust (sandwich) covariance estimators were used for the regression coefficients. The factors, *stimulation* (cerebellum, TPJ, SMA, sham), *test modality* (auditory, visual), *test delay* (0, 83, 167, 250, 333, 417ms), and *group* (HC, SSD) were included in a full factorial model testing for all main and interaction effects. The active-passive difference was modeled with a linear link function. Importantly, this analysis revealed a significant interaction of *group* and *test delay* [Wald Chi-Square (df = 5) = 13.332, *p* = .020]. According to post-hoc tests, the active-passive difference was significantly stronger in HC than in SSD for test stimuli delayed by 250ms [mean difference = 11.512, standard error = 3.928, df = 1, *p* = .003].

Hence, for an individual medium-sized delay level, patients showed reduced differences in the perception of actively vs. passively elicited stimuli. Importantly, the medium-sized delay levels are the ones for which most prominent active-passive differences can be assumed due to floor or ceiling effects at very small or large delays, respectively. Thus, to a given extent, there are indications for the aberrant processing of actively generated action-outcomes in SSD in in our study, but we cannot provide evidence for the attribution of this impairment to dysfunctional temporal recalibration mechanisms.

**S6. Exploratory comparisons between effects of the three active stimulation conditions on the TRE**

We also explored whether the TRE with cerebellar tDCS, particularly in active and auditory conditions, was not only significantly larger compared to sham stimulation (as reported in the main manuscript), but whether it was also significantly larger compared to tDCS of the TPJ or SMA. To this end, by means of one-sided two-samples t-tests, we compared the TRE in these conditions between cerebellar tDCS and the other two active tDCS conditions.

Across groups, the TRE was significantly larger with cerebellar tDCS compared to tDCS of the TPJ [Across groups: Mean difference = 3.983, *SD* = 13.713, *t*(41) = 1.960, *p* = .028, *d* = .302; SSD: Mean difference = 3.538, *SD* = 18.248, *t*(21) = .909, *p* = .187, *d* = .194; HC: Mean difference = 4.473, *SD* = 9.928, *t*(19) = 2.015, *p* = .029, *d* = .451]. Similarly, across patients and HC, the TRE was significantly larger with cerebellar tDCS compared to tDCS of the SMA [Across groups: Mean difference = 4.164, *SD* = 15.541, *t*(41) = 1.736, *p* = .045, *d* = .268; SSD: Mean difference = 4.764, *SD* = 19.575, *t*(21) = 1.141, *p* = .133, *d* = .243; HC: Mean difference = 3.503, *SD* = 12.095, *t*(19) = 1.295, *p* = .105, *d* = .290]. Furthermore, according to a two-sided, two-samples t-test, the TRE did not differ significantly between TPJ and SMA stimulation [Across groups: Mean difference = .180, *SD* = 11.268, *t*(41) = .104, *p* = .918, *d* = .016; SSD: Mean difference = 1.226, *SD* = 14.588, *t*(21) = .394, *p* = .698, *d* = .084; HC: Mean
difference = -.970, *SD* = 9.769, *t*(19) = -.444, *p* = .662, *d* = -.099].

Thus, the effects across both groups indicate the superiority of cerebellar tDCS in facilitating the TRE not only in comparison to sham stimulation but also in comparison to tDCS of the TPJ and SMA. It must be noted though that the tests for each individual group seemed to have lacked sufficient statistical power to consistently reveal significant differences between stimulation conditions. Nonetheless, these results provide further evidence for the importance specifically of the cerebellum in the recalibration of forward model predictions, as compared to the TPJ and SMA which were also frequently associated with processes related to the forward model, such as action-outcome processing and the sense of agency. Furthermore, they emphasize that the cerebellum may be the most promising stimulation site for enhancing the adaptability of forward model predictions.

**S7. Exploratory analysis of patient subgroups**

The SSD group in our study did not only consist of patients with a F20 diagnosis of schizophrenia (SZ) but also included a small group of patients diagnosed with a schizoaffective disorder (SZA). To test whether the results reported in the main manuscript are driven by the SZA group and do not reflect the pattern in the SZ group, the main analyses were exploratorily performed for the subgroup of patients with SZ (N = 15). Since our sample only comprised 6 patients with SZA, the data pattern for this group was inspected descriptively only.

Results of all main and interaction effects of the mixed ANOVA with the factors *group* (SZ, HC), *movement type* (active, passive), *test modality* (visual, auditory), and *adaptation delay* (0ms, 200ms) are depicted in **Supplementary Table 4** and in **Supplementary Fig. 1**. An overview of effects computed individually for the SZ group are additionally provided in **Supplementary Table 5**. As in the analysis with the entire SSD sample reported in the main manuscript, there was no significant main effect of *group* [*F*(1, 33) = .010, *p* = .922, η_p_^2^ < .001] and no interaction effects including the factors *group* and *adaptation delay* (all *p* > .068). Across groups and conditions, there was a significant main effect of the *adaptation delay*
[*F*(1, 33) = 13.934, *p* < .001, η_p_^2^ = .297], indicating significant temporal recalibration [*Mean TRE* = 3.334, *SD* = 5.319]. The significant interaction of *movement type* and *adaptation delay*
[*F*(1, 33) = 14.242, *p* = < .001, η_p_^2^ = 0.301] revealed that the TRE was significantly greater than zero in both active [*Mean TRE* = 4.647, *SD* = 4.390, *t*(34) = 6.263, *p* < .001, *d* = 1.059,
ɑ_corr_ = .025] and passive conditions [*Mean TRE* = 2.020, *SD* = 6.715, *t*(34) = 1.780, *p* = .042,
*d* = .301, ɑ_corr_ = .025], but was significantly stronger in active ones [*Mean difference* = 2.627,
*SD* = 3.944, *t*(34) = 3.940, *p* < .001, *d* = .666]. According to the significant *test modality* and *adaptation delay* interaction [*F*(1, 33) = 7.293, *p* = .011, η_p_^2^ = .181], the TRE was significantly greater than zero for both, audition [*Mean TRE* = 4.366, *SD* = 5.730, *t*(34) = 4.508,
*p* < .001, *d* = .762, ɑ_corr_ = .025] and vision [*Mean TRE* = 2.301, *SD* = 5.734, *t*(34) = 2.374,
*p* = .012, *d* = .401, ɑ_corr_ = .025], but remained significantly larger in auditory (unimodal) than in visual (cross-modal) conditions [*Mean difference* = 2.065, *SD* = 4.273, *t*(34) = 2.859, *p* = .004, *d* = .483]. The significant interaction of the three factors *movement type*, *test modality*, and *adaptation delay* [*F*(1, 33) = 5.265, *p* = .028, η_p_^2^ = .138] further indicated that the active-passive difference in the TRE was specific to auditory outcomes [*Mean difference* = 4.662,
*SD* = 6.442, *t*(34) = 4.281, *p* < .001, *d* = .724, ɑ_corr_ = .025; Active: *Mean TRE* = 6.698,
*SD* = 6.120, *t*(34) = 6.474, *p* < .001, *d* = 1.094, ɑ_corr_ = .025; Passive: *Mean TRE* = 2.035,
*SD* = 6.997, *t*(34) = 1.721, *p* = .047, *d* = .291, ɑ_corr_ = .025] but did not transfer to the visual modality [*Mean difference* = .592, *SD* = 6.641, *t*(34) = .527, *p* = .301, *d* = .089, ɑ_corr_ = .025; Active: *Mean TRE* = 2.597, *SD* = 4.495, *t*(34) = 3.418, *p* = < .001, *d* = .578, ɑ_corr_ = .025; Passive: *Mean TRE* = 2.005, *SD* = 8.223, *t*(34) = 1.443, *p* = .079, *d* = .244, ɑ_corr_ = .025].

Regarding stimulation dependent effects, the significant interaction of *stimulation* and *adaptation delay* [*F*(3, 99) = 3.333, *p* = .023, η_p_^2^ = .092] revealed that the TRE was significantly stronger after cerebellar tDCS compared to sham stimulation [*Mean difference* = 3.772,
*SD* = 7.892, *t*(34) = 2.827, *p* = .004, *d* = .478, ɑ_corr_ = .016; Sham: *Mean TRE* = 1.692,
*SD* = 6.476, *t*(34) = 1.545, *p* = .066, *d* = .401, ɑ_corr_ = .025; Cerebellum: *Mean TRE* = 5.463,
*SD* = 7.750, *t*(34) = 4.171, *p* < .001, *d* = .705, ɑ_corr_ = .025], but not after tDCS on the right SMA [*Mean difference* = 1.012, *SD* = 8.645, *t*(34) = .693, *p* = .247, *d* = .117, ɑ_corr_ = .016] or the right TPJ [*Mean difference* = 1.784, *SD* = 6.244, *t*(34) = 1.691, *p* = .050, *d* = .286, ɑ_corr_ = .016]. Contrary to the analysis reported in the main manuscript, the four-way interaction of *stimulation*, *movement type*, *test modality*, and *adaptation delay* did not reach significance [*F*(3, 99) = .232, *p* = .874, η_p_^2^ = .007].

Overall, the results of this analysis limited to the subgroup of patients with SZ show strong similarities to the results reported with the entire SSD sample in the main manuscript, with comparable TREs for patients and HC, and a faciliatory impact of cerebellar tDCS on the TRE. Minor differences between the analyses, such as the non-significant four-way interaction when only including SZ patients, may be attributed to the smaller sample size and thus reduced statistical power in the SZ subgroup. Furthermore, although the number of SZA patients in our sample was very small, the pattern of results in this subgroup also aligns with that of the SZ subgroup and the combined SSD group (see **Supplementary Fig. 2**). These results could suggest that the underlying temporal recalibration mechanisms and cerebellar processes are comparable for different diagnoses of the schizophrenia spectrum. This is in line with previous findings demonstrating that similar deficits in processing self-generated action-outcomes are associated with SZ and SZA,^14^ indicating commonalities in sensorimotor functions and their impairments in psychosis with and without affective symptomatology. However, it is important to emphasize that due to the small sample sizes in the patient subgroups in the present study, conclusions about the subgroup results should be taken with caution.

**Supplementary Table 4. Results of the ANOVA testing for differences in delay detection performances between the experimental conditions only including the subgroup of patients diagnosed with a F20 diagnosis of schizophrenia.**

| **Effect** | **Sum of Squares** | **df** | **Mean Square** | **F-val.** | **p-val.** | **η_p_^2^** |  |
| --- | --- | --- | --- | --- | --- | --- | --- |
| **Group** | 36.626 | 1 | 36.626 | 0.010 | 0.922 | 2.970e -4 |  |
| Residuals | 123266.193 | 33 | 3735.339 |  |  |  |  |
| **Stimulation** | 2345.506 | 3 | 781.835 | 4.064 | 0.009 | 0.110 |  |
| **Stimulation * Group** | 716.295 | 3 | 238.765 | 1.241 | 0.299 | 0.036 |  |
| Residuals | 19045.700 | 99 | 192.381 |  |  |  |  |
| **Test modality** | 665.489 | 1 | 665.489 | 3.133 | 0.086 | 0.087 |  |
| **Test modality * Group** | 117.060 | 1 | 117.060 | 0.551 | 0.463 | 0.016 |  |
| Residuals | 7009.874 | 33 | 212.420 |  |  |  |  |
| **Movement type** | 8353.120 | 1 | 8353.120 | 37.985 | < .001 | 0.535 |  |
| **Movement type * Group** | 106.324 | 1 | 106.324 | 0.483 | 0.492 | 0.014 |  |
| Residuals | 7256.944 | 33 | 219.907 |  |  |  |  |
| **Adaptation delay** | 3207.381 | 1 | 3207.381 | 13.934 | < .001 | 0.297 |  |
| **Adaptation delay * Group** | 98.913 | 1 | 98.913 | 0.430 | 0.517 | 0.013 |  |
| Residuals | 7596.323 | 33 | 230.192 |  |  |  |  |
| **Stimulation * Test modality** | 112.140 | 3 | 37.380 | 1.259 | 0.293 | 0.037 |  |
| **Stimulation * Test modality * Group** | 21.372 | 3 | 7.124 | 0.240 | 0.868 | 0.007 |  |
| Residuals | 2939.090 | 99 | 29.688 |  |  |  |  |
| **Stimulation * Movement type** | 231.092 | 3 | 77.031 | 1.363 | 0.259 | 0.040 |  |
| **Stimulation * Movement type * Group** | 39.864 | 3 | 13.288 | 0.235 | 0.872 | 0.007 |  |
| Residuals | 5595.882 | 99 | 56.524 |  |  |  |  |
| **Test modality * Movement type** | 2520.472 | 1 | 2520.472 | 62.251 | < .001 | 0.654 |  |
| **Test modality * Movement type * Group** | 11.838 | 1 | 11.838 | 0.292 | 0.592 | 0.009 |  |
| Residuals | 1336.136 | 33 | 40.489 |  |  |  |  |
| **Stimulation * Adaptation delay** | 551.740 | 3 | 183.913 | 3.333 | 0.023 | 0.092 |  |
| **Stimulation * Adaptation delay * Group** | 134.200 | 3 | 44.733 | 0.811 | 0.491 | 0.024 |  |
| Residuals | 5462.450 | 99 | 55.176 |  |  |  |  |
| **Test modality * Adaptation delay** | 269.260 | 1 | 269.260 | 7.293 | 0.011 | 0.181 |  |
| **Test modality * Adaptation delay * Group** | 23.427 | 1 | 23.427 | 0.635 | 0.431 | 0.019 |  |
| Residuals | 1218.355 | 33 | 36.920 |  |  |  |  |
| **Movement type * Adaptation delay** | 412.343 | 1 | 412.343 | 14.242 | < .001 | 0.301 |  |
| **Movement type * Adaptation delay * Group** | 102.564 | 1 | 102.564 | 3.543 | 0.069 | 0.097 |  |
| Residuals | 955.419 | 33 | 28.952 |  |  |  |  |
| **Stimulation * Test modality * Movement type** | 37.519 | 3 | 12.506 | 0.637 | 0.593 | 0.019 |  |
| **Stimulation * Test modality * Movement type * Group** | 28.846 | 3 | 9.615 | 0.490 | 0.690 | 0.015 |  |
| Residuals | 1944.185 | 99 | 19.638 |  |  |  |  |
| **Stimulation * Test modality * Adaptation delay** | 138.749 | 3 | 46.250 | 1.814 | 0.149 | 0.052 |  |
| **Stimulation * Test modality * Adaptation delay * Group** | 8.956 | 3 | 2.985 | 0.117 | 0.950 | 0.004 |  |
| Residuals | 2523.582 | 99 | 25.491 |  |  |  |  |
| **Stimulation * Movement type * Adaptation delay** | 101.036 | 3 | 33.679 | 0.951 | 0.419 | 0.028 |  |
| **Stimulation * Movement type * Adaptation delay * Group** | 3.637 | 3 | 1.212 | 0.034 | 0.991 | 0.001 |  |
| Residuals | 3504.506 | 99 | 35.399 |  |  |  |  |
| **Test modality * Movement type * Adaptation delay** | 294.782 |  | 294.782 | 5.265 | 0.028 | 0.138 |  |
| **Test modality * Movement type * Adaptation delay * Group** | 4.851 | 1 | 4.851 | 0.087 | 0.770 | 0.003 |  |
| Residuals | 1847.790 | 33 | 55.994 |  |  |  |  |
| **Stimulation * Test modality * Movement type * Adaptation delay** | 138.584 | 3 | 46.195 | 2.176 | 0.096 | 0.062 |  |
| **Stimulation * Test modality * Movement type * Adaptation delay * Group** | 14.775 | 3 | 4.925 | 0.232 | 0.874 | 0.007 |  |
| Residuals | 2101.705 | 99 | 21.229 |  |  |  |  |

*Note.* N_HC_ = 20, N_SZ_ = 15

**
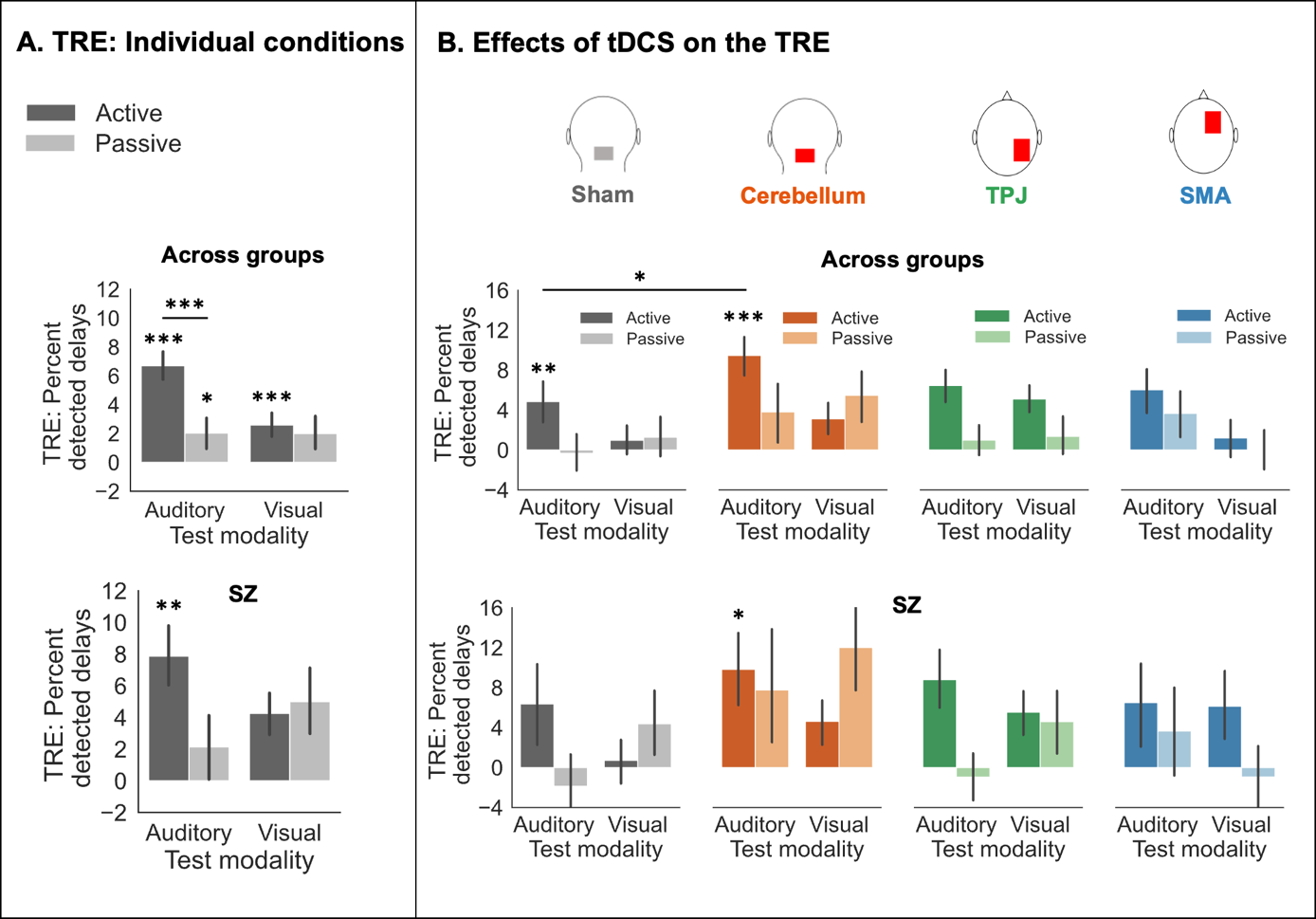
**

**Supplementary Fig. 1. Temporal recalibration effects for the patient subgroup with schizophrenia.** **A:** The TRE, defined as the difference in the percentage of detected delays between conditions with the 200ms vs. 0ms delay during preceding adaptation phases, is displayed for each experimental condition (i.e., for both test modalities and movement types), across HC and patients diagnosed with a F20 diagnosis of schizophrenia (SZ; N = 15), as well as separately for SZ patients. **B:** The TRE is displayed for each of the four stimulation conditions, again across HC and SZ patients and separately for SZ patients. Error bars indicate standard errors of the mean. **p* > .05,
***p* < .01, ****p* < .001.

**Supplementary Table 5. TREs for individual conditions and comparisons of conditions, evaluated for the subgroup of patients diagnosed with a F20 diagnosis of schizophrenia.**

| **TRE** | **Mean+/-SD** | **t-value** | **p-value** | **ɑ_corr_** | **Cohen´s d** |
| --- | --- | --- | --- | --- | --- |
| Across conditions | **4.821 +/- 8.199** | **2.278** | **.019*** | **.05** | **.588** |
| Active | **6.064 +/ 8.207** | **2.862** | **.006***** | **.025** | **.739** |
| Passive | 3.578 +/- 9.160 | 1.513 | .076 | .025 | .391 |
| Active > Passive | 2.486 +/- 5.802 | 1.659 | .060 | .05 | .428 |
| Auditory | 5.010 +/- 9.122 | 2.127 | .026 | .025 | .549 |
| Visual | 4.632 +/- 8.439 | 2.126 | .026 | .025 | .549 |
| Auditory > Visual | .378 +/- 6.323 | .231 | .410 | .05 | .060 |
| Auditory: Active | **7.872 +/- 11.416** | **2.671** | **.009**** | **.025** | **.690** |
| Auditory: Passive | 2.148 +/- 9.598 | .867 | .200 | .025 | .224 |
| Visual: Active | **4.256 +/- 7.284** | **2.263** | **.020*** | **.025** | **.584** |
| Visual: Passive | 5.009 +/- 10.618 | 1.827 | .045 | .025 | .472 |
| Auditory:  Active > Passive | **5.724 +/- 10.586** | **2.094** | **.027** | **.025** | **.541** |
| Visual:  Active > Passive | -.752 +/- 6.836 | -.426 | .662 | .025 | -.110 |
| Sham | 2.401 +/- 8.866 | 1.049 | .156 | .025 | .271 |
| Cerebellum | **8.554 +/- 12.707** | **2.607** | **.010**** | **.025** | **.673** |
| Cerebellum > Sham |  | **2.116** | **.026*** | **.05** | **.546** |
| Active/Auditory:  Sham | 6.372 +/- 16.801 | 1.469 | .082 | .025 | .379 |
| Active/Auditory:  Cerebellum | **9.832 +/- 15.441** | **2.466** | **.014*** | **.025** | **.637** |
| Active/Auditory:  Cerebellum > Sham | 3.461 +/- 18.389 | .729 | .239 | .05 | .188 |

*Note*. The TRE is defined as the difference in the percentage of detected delays between conditions with the 200ms vs. 0ms delay during preceding adaptation phases. For individual conditions, one-sample t-tests were used to assess whether the TRE was significantly greater than zero. Difference in TRE between conditions were assessed with two-samples t-tests. All t-tests were Bonferroni corrected. The corrected alpha level used for each test is displayed in the column ɑ_corr_. Significant tests are presented with bold values. N_SZ_ = 15. **p* < .05, ***p* < .01,
****p* < .001

**
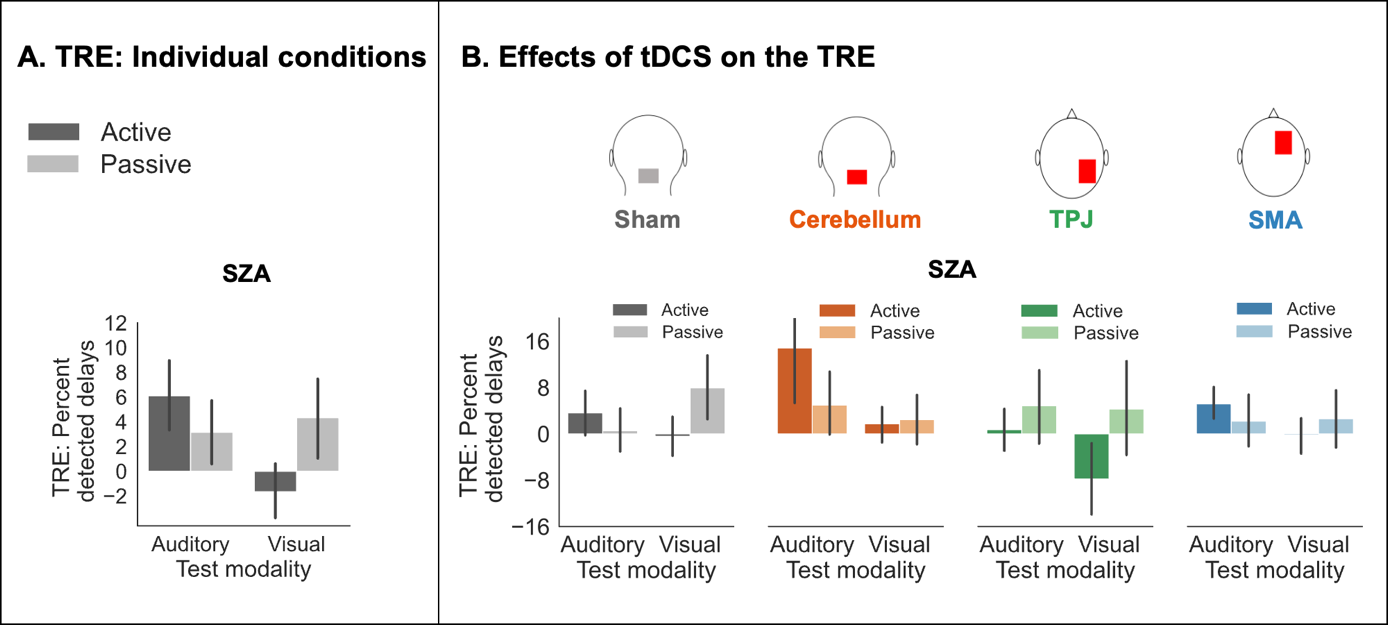
**

**Supplementary Fig. 2. Temporal recalibration effects for the patient subgroup with schizoaffective disorder.** **A:** The TRE, defined as the difference in the percentage of detected delays between conditions with the 200ms vs. 0ms delay during preceding adaptation phases, is displayed for each experimental condition (i.e., for both test modalities and movement types) for the subgroup of patients diagnosed with schizoaffective disorder (SZA;
N = 6). **B:** The TRE is displayed for each of the four stimulation conditions. Error bars indicate standard errors of the mean.

**S8. Limitations**

The results of our study do not provide evidence for impaired sensorimotor temporal recalibration mechanisms in patients with SSD, as there were no significant differences compared to the HC group. This could indicate that sensorimotor recalibration abilities may be a useful resource of patients with SSD that could be exploited to train predictive mechanisms based on the forward model and to thereby improve self-other differentiation and action-outcome monitoring. Importantly, however, the absence of group differences in recalibration does not necessarily speak against the existence of an impairment in patients; it is also conceivable that certain characteristics of our study have masked differences between the groups.

Firstly, previous studies on potentially related adaptive processes, namely on sensorimotor adaptation, could show that patients were able to adapt their movements to the introduced action feedback perturbations, but they adapted slower^15^ and the adaptation process was associated with more errors^16^ compared to HC. Our study design did not allow for the investigation of the time course of temporal recalibration effects. Adaptation and test phases were blocked, which only allowed us to assess the impact of the adaptation delay on perception once at the end of an adaptation phase. Thus, future study designs should consider that the time course of adaptation could provide important information regarding potential impairments in patients.

Secondly, the majority of patients in our sample were under antipsychotic medication at the time of the study. This could have compensated for potentially existing deficits, since antipsychotics are known to particularly target positive symptoms, such as hallucinations and delusions, which are believed to be associated with the dysfunctions in predictive mechanisms of the forward model investigated here.^10–13,17,18^ Additionally, the patients exhibited on average a relatively high level of functioning and displayed only moderate levels of symptoms at the time of testing, as indicated by the SANS and SAPS scores (see Supplementary Table 1). Therefore, it should be noted that it remains open whether group differences in sensorimotor temporal recalibration might have emerged in a sample characterized by higher average symptom scores and a larger number of patients. Although our data indicate that recalibration processes are comparable between patients and healthy individuals, cerebellar tDCS may also have the potential to normalize patients´ recalibration performance if they exhibit impairments in this process.

Thirdly, the variance in temporal recalibration and stimulation effects appeared to be high in our study, particularly in the patient group. While this may partly be explained by the relatively low sample sizes, it could also suggest that there are individual differences in whether patients exhibit dysfunctions in sensorimotor temporal recalibration mechanisms. Likewise, there could be individual differences in the effectiveness of tDCS on enhancing recalibration effects. Hence, it would be useful to determine under which conditions patients may exhibit dysfunctions in temporal recalibration or profit from tDCS. A relevant factor could, for instance, be the presence the above-mentioned symptoms related to the presumed deficits in predictive mechanisms. In our study, exploratory correlation analyses did not reveal a relationship between temporal recalibration and these symptoms (see supplementary material S9), but future studies with larger sample sizes could specifically investigate the determining factors for the occurrence of a potential deficit in this process and the effectiveness of tDCS on facilitating the underlying predictive mechanisms. Furthermore, variability in stimulation-dependent effects may also arise due to individual differences in participants’ cerebellar anatomy and connectivity pattern with other brain regions.^19^ Thus, future studies could apply individually adjusted stimulation protocols according to the participants’ individual anatomy.

Finally, it should be acknowledged that the actual mechanism underlying sensorimotor temporal recalibration are not yet fully understood. A recent and ongoing debate has cast doubt on the suitability of the forward model framework for explaining the processing of all types of action-outcomes. It has been suggested that external outcomes of an action which are not body-related, such as the abstract visual and auditory stimuli employed in the present study, may be subject to more general predictive mechanisms that operate across perceptual and motor domains, as opposed to forward model predictions based on efference copy signals.^20–22^ Importantly though, the difference in the TRE between active and passive movement conditions observed here as well as in previous studies^23–26^ indicates that there are unique characteristics in predictive mechanisms depending on whether an active action is involved, as proposed by the forward model framework. Nonetheless, the exact neural mechanisms by which action-outcome predictions are generated and recalibrated in regions like the cerebellum remain to be more closely examined.

**S9. Exploratory correlation analyses of the TRE and SAPS score**

Dysfunctions in predictive mechanisms of the forward model, i.e., in adequately predicting the sensory outcomes of self-generated actions, are assumed to partly underly symptoms in SSD, such as hallucinations (e.g., perceiving the own inner speech as external voice) and ego-disturbances or delusions of control (e.g., perceiving own thoughts or actions as externally controlled).^27^ Thus, it is conceivable that the severity of these symptoms correlates with the ability to recalibrate forward model predictions in response to changes in environmental conditions. To test this, we performed exploratory correlation analyses between the TRE and the total SAPS score as well as the SAPS subscales for hallucinations and delusions obtained from the patients. Furthermore, we tested whether the amount of facilitation of the TRE by cerebellar tDCS correlated with the same clinical measures. All correlations were performed with the SciPy package (version 1.11.1) for Python (version 3.11; <https://www.python.org/>). However, none of these correlations reached significance (see **Supplementary Fig. 3**). This could either indicate that the emergence of the TRE did not depend on the severity of the respective symptoms in our study but may also be explained by the relatively small sample of 22 patients, which might have resulted in insufficient statistical power for such effects to emerge (see also supplementary material S8).

**
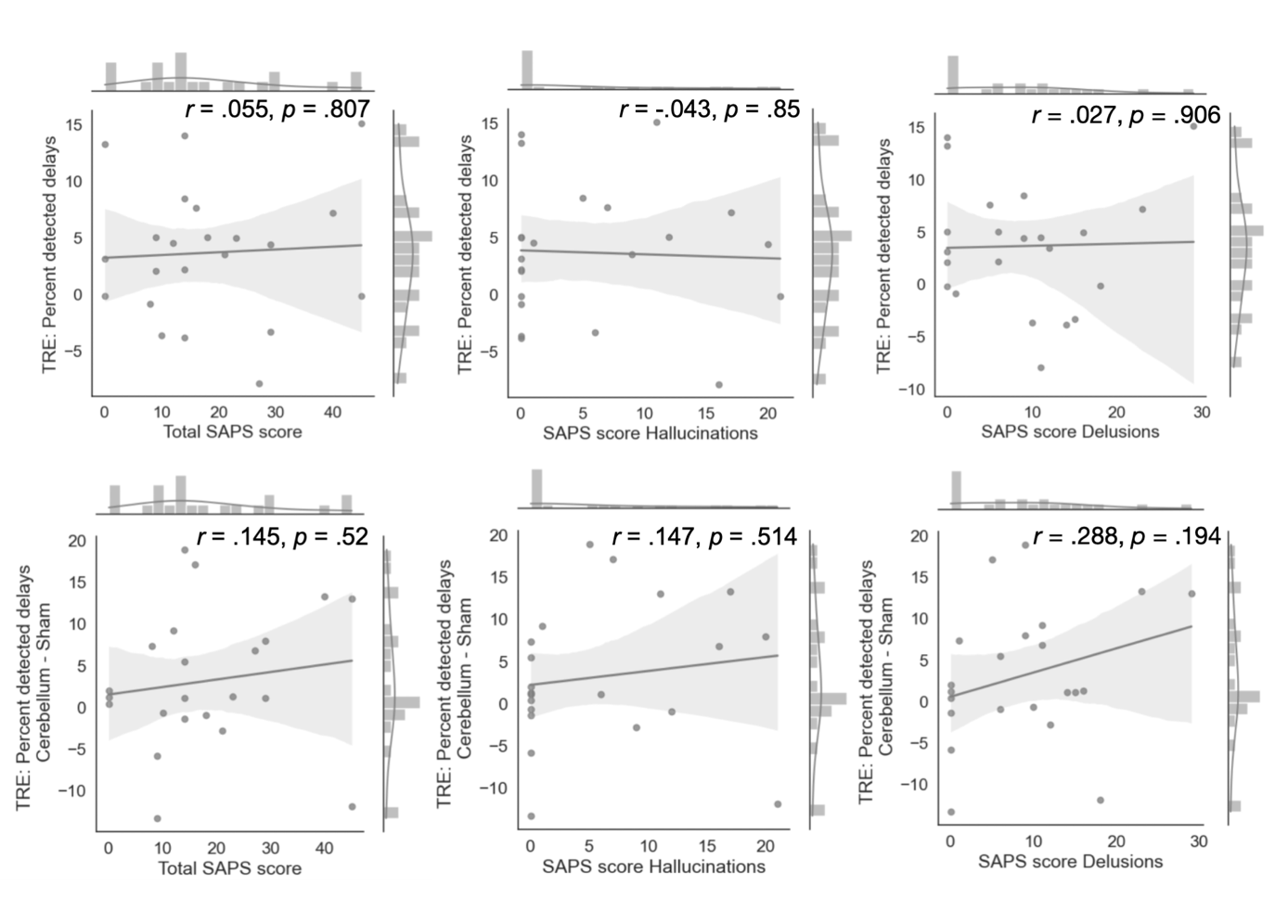
**

**Supplementary Fig. 3. Upper row:** Correlations between the TRE and the SAPS score (total score and for the subscales on hallucinations and delusions) of the SSD group are displayed. **Lower row:** Correlations are displayed between the same SAPS scores and the amount of facilitation of the TRE by cerebellar tDCS compared to sham stimulation. ­**References**

1. First MB, Gibbon M, Spitzer RL, Williams JBW, Benjamin LS. *Structured Clinical Interview for DSM-IV Axis II Personality Disorders, (SCID-II).* Washington, DC: American Psychiatric Association; 1997.

2. Brickenkamp R. *D2 Aufmerksamkeits-Belastungs-Test.* 8th ed. Göttingen: Hogrefe; 1994.

3. Reitan RM. *Trail Making Test*. Tucson, AZ: Reitan Neuropsychology Laboratory; 1992.

4. Petermann F. *WAIS-IV. Wechsler Adult Intelligence Scale*. Frankfurt: Pearson; 2012.

5. Andreasen NC. *The Scale for the Assessment of Positive Symptoms (SAPS)*. Iowa City: The University of Iowa; 1984.

6. Andreasen NC. *The Scale for the Assessment of Negative Symptoms (SANS)*. Iowa City: The University of Iowa; 1983.

7. Beck AT, Steer RA, Brown G. *Beck Depression Inventory–II (BDI-II)*. APA PsycTests; 1996.

8. Hall RCW. Global Assessment of Functioning. *Psychosomatics*. 1995;36(3):267-275. doi:10.1016/S0033-3182(95)71666-8

9. Goldman HH, Skodol AE, Lave TR. Revising axis V for DSM-IV: a review of measures of social functioning. *American Journal of Psychiatry*. 1992;149(9):1148-1156. doi:10.1176/ajp.149.9.1148

10. Ford JM, Mathalon DH, Heinks T, Kalba S, Faustman WO, Roth WT. Neurophysiological Evidence of Corollary Discharge Dysfunction in Schizophrenia. *American Journal of Psychiatry*. 2001;158(12):2069-2071. doi:10.1176/appi.ajp.158.12.2069

11. Martinelli C, Rigoli F, Shergill SS. Aberrant Force Processing in Schizophrenia. *Schizophrenia Bulletin*. 2016;(2):417-424. doi:10.1093/schbul/sbw092

12. Shergill SS, White TP, Joyce DW, Bays PM, Wolpert DM, Frith CD. Functional Magnetic Resonance Imaging of Impaired Sensory Prediction in Schizophrenia. *JAMA Psychiatry*. 2014;71(1):28. doi:10.1001/jamapsychiatry.2013.2974

13. Uhlmann L, Pazen M, van Kemenade BM, Kircher T, Straube B. Neural Correlates of Self-other Distinction in Patients with Schizophrenia Spectrum Disorders: The Roles of Agency and Hand Identity. *Schizophrenia Bulletin*. 2021;47(5):1399-1408. doi:10.1093/schbul/sbaa186

14. Ford JM, Mathalon DH, Roach BJ, et al. Neurophysiological Evidence of Corollary Discharge Function During Vocalization in Psychotic Patients and Their Nonpsychotic First-Degree Relatives. *Schizophrenia Bulletin*. 2013;39(6):1272-1280. doi:10.1093/schbul/sbs129

15. Coesmans M, Röder C, Smit A, et al. Cerebellar motor learning deficits in medicated and medication-free men with recent-onset schizophrenia. *Journal of Psychiatry & Neuroscience*. 2014;39(1):E3-E11. doi:10.1503/jpn.120205

16. Cornelis C, De Picker LJ, Coppens V, et al. Impaired Sensorimotor Adaption in Schizophrenia in Comparison to Age-Matched and Elderly Controls. *Neuropsychobiology*. 2022;81(2):127-140. doi:10.1159/000518867

17. Lindner A, Thier P, Kircher TTJ, Haarmeier T, Leube DT. Disorders of Agency in Schizophrenia Correlate with an Inability to Compensate for the Sensory Consequences of Actions. *Current Biology*. 2005;15(12):1119-1124. doi:10.1016/j.cub.2005.05.049

18. Frith CD, Blakemore SJ, Wolpert DM. Abnormalities in the awareness and control of action. *Philosophical Transactions of the Royal Society of London Series B: Biological Sciences*. 2000;355(1404):1771-1788. doi:10.1098/rstb.2000.0734

19. Tzvi E, Loens S, Donchin O. Mini-review: The Role of the Cerebellum in Visuomotor Adaptation. *Cerebellum*. 2022;21(2):306-313. doi:10.1007/s12311-021-01281-4

20. Dogge M, Custers R, Aarts H. Moving Forward: On the Limits of Motor-Based Forward Models. *Trends in Cognitive Sciences*. 2019;23(9):743-753. doi:10.1016/j.tics.2019.06.008

21. Jagini KK. Temporal Binding in Multisensory and Motor-Sensory Contexts: Toward a Unified Model. *Front Hum Neurosci*. 2021;15:629437. doi:10.3389/fnhum.2021.629437

22. Press C, Kok P, Yon D. The Perceptual Prediction Paradox. *Trends in Cognitive Sciences*. 2020;24(1):13-24. doi:10.1016/j.tics.2019.11.003

23. Arikan BE, van Kemenade BM, Fiehler K, Kircher T, Drewing K, Straube B. Different contributions of efferent and reafferent feedback to sensorimotor temporal recalibration. *Scientific Reports*. 2021;11(1):22631. doi:10.1038/s41598-021-02016-5

24. Stetson C, Cui X, Montague PR, Eagleman DM. Motor-Sensory Recalibration Leads to an Illusory Reversal of Action and Sensation. *Neuron*. 2006;51(5):651-659. doi:10.1016/j.neuron.2006.08.006

25. Schmitter CV, Kufer K, Steinsträter O, Sommer J, Kircher T, Straube B. Neural correlates of temporal recalibration to delayed auditory feedback of active and passive movements. *Human Brain Mapping*. Published online October 11, 2023:hbm.26508. doi:10.1002/hbm.26508

26. Schmitter CV, Straube B. The impact of cerebellar transcranial direct current stimulation (tDCS) on sensorimotor and inter-sensory temporal recalibration. *Frontiers in Human Neuroscience*. 2022;16:998843. doi:10.3389/fnhum.2022.998843

27. Pynn LK, DeSouza JFX. The function of efference copy signals: Implications for symptoms of schizophrenia. *Vision Research*. 2013;76:124-133. doi:10.1016/j.visres.2012.10.019
